# Evidence-Based and Emerging Diet Recommendations for Small Bowel Disorders

**DOI:** 10.14309/ajg.0000000000001764

**Published:** 2022-04-08

**Authors:** Anupam Rej, Michael D. E. Potter, Nicholas J. Talley, Ayesha Shah, Gerald Holtmann, David Surendran Sanders

**Affiliations:** 1 Academic Unit of Gastroenterology, Royal Hallamshire Hospital, Sheffield Teaching Hospital NHS Foundation Trust, Sheffield, England;; 2 Faculty of Health and Medicine, University of Newcastle, Newcastle, New South Wales, Australia;; 3 Faculty of Medicine and Faculty of Health and Behavioral Sciences, The University of Queensland, Brisbane, Australia;; 4 Department of Gastroenterology and Hepatology, Princess Alexandra Hospital, Brisbane, Australia;; 5 AGIRA (Australian Gastrointestinal Research Alliance) and the NHMRC Centre of Research Excellence in Digestive Health, Brisbane, Australia;; 6 Academic Unit of Gastroenterology, Department of Infection, Immunity, and Cardiovascular Disease, University of Sheffield, Sheffield, United Kingdom.

## Abstract

Diet plays a key role in the manifestation and severity of gastrointestinal symptoms, with increasing research interest on the role of diet in small bowel disorders. There are predominantly 3 small bowel conditions that have potential dietary interventions. Self-reported nonceliac gluten/wheat sensitivity is prevalent. Although gluten is believed to be a potential trigger for symptoms, other components of wheat may also be triggers, including fructans, alpha-amylase trypsin inhibitors, and wheat germ agglutinins. The diagnosis can be challenging, given the lack of validated biomarkers. A gluten-free diet that excludes the abovementioned triggers is the cornerstone of treatment; however, unlike celiac disease, there is uncertainty about the level of adherence or whether the gluten-free diet is a lifelong intervention. Several primary gastrointestinal disorders are associated with an increase in inflammatory cells including eosinophils. Diet seems to be an important driver of disease pathogenesis in eosinophilic gastroenteritis, with elimination and elemental diets showing promise in management, with further robust trials required. Small intestinal bacterial overgrowth is an example of microbial dysbiosis, with renewed interest in diet being postulated to cause an adaptive change of the microbes colonizing the small intestine. However, the diagnosis of small intestinal bacterial overgrowth is limited by a lack of sensitive and specific tests, with significant knowledge gaps in relation to therapeutic measures to manage and cure small intestinal bacterial overgrowth. Currently, antimicrobials are the established management option. There have been significant clinical advances in dietary interventions related to the small bowel, but this area is currently a novel and advancing field for both patients and clinicians.

## INTRODUCTION

The composition of our diet seems to be key for the manifestation of gastrointestinal symptoms, playing a key role in common small bowel conditions, such as nonceliac gluten/wheat sensitivity (NCG/WS) (1), and being important in rarer conditions, such as eosinophilic gastroenteritis (EGE) (2,3). In addition, diet may result in alterations in the gut microbiome (4), having a potential role in the management of small intestinal bacterial overgrowth (SIBO). The aim of this article is to summarize the emerging knowledge on the role of dietary stimuli in small bowel disorders.

## NONCELIAC GLUTEN/WHEAT SENSITIVITY

### Key concepts

NCG/WS was first coined in the late 1970s (5) and is characterized by symptoms triggered by the ingestion of gluten or wheat products, with individuals presenting with intestinal and extraintestinal manifestations, in the context of celiac disease and wheat allergy being excluded (6).

The reported prevalence of NCG/WS ranges between 0.49% and 14.9% in the published literature (7). The variable prevalence rate is in part due to NCG/WS being self-reported, differing population groups, and there being a lack of diagnostic biomarkers for its diagnosis. Formalized criteria for its diagnosis, using the Salerno's experts criteria, have been developed, involving assessing response to a gluten-free diet (GFD) and measuring the effect of reintroduction of gluten after a period of being on a GFD (6). However, it is worth noting that this is rarely applied outside research settings, with many patients already on a GFD and unwilling to reintroduce gluten at time of diagnosis (6). A more pragmatic approach of assessing symptoms on a gluten-containing diet vs a GFD has been suggested for diagnosis (8). The controversy is that currently systematic review and meta-analysis suggest that the worldwide prevalence of celiac disease is around 1% (9), and most cases remain unrecognized. This means that some patients with self-reported NCG/WS may have undiagnosed celiac disease. If these patients are on a GFD, they should be encouraged to ensure that they do not have celiac disease by undergoing a gluten challenge along with appropriate diagnostic testing (10).

The pathophysiology of NCG/WS is still not fully understood. Although wheat has been postulated as being the key trigger for symptoms, the component of wheat that triggers symptoms in individuals currently seems to be unclear. Several components of wheat have been postulated as key for symptom generation in NCG/WS, including gluten, fructans (a FODMAP), wheat germ agglutinins, alpha-amylase trypsin inhibitors, and potentially a nocebo response.

NCG/WS seems to be triggered by activation of the innate immune system rather than the adaptive immune system. This is because markers such as toll-like receptor 2 have been shown to be increased in NCG/WS consistent with innate immunity, with adaptive markers such as interleukin-6 and interleukin-21 not being expressed at high levels (11). However, there may also be a role for the adaptive immune system with an increase in interferon-γ being noted in NCG/WS (12). This has also been noted in individuals with irritable bowel syndrome (IBS) (13), highlighting that this may not be specific to NCG/WS (14), with an overlap of NCG/WS and IBS being suggested in the literature (15). Systemic immune activation has been suggested in NCG/WS with an increase in serum levels of CD14, lipopolysaccharide-binding protein, and antibacterial antibodies observed (14,16).

**Figure 1.**
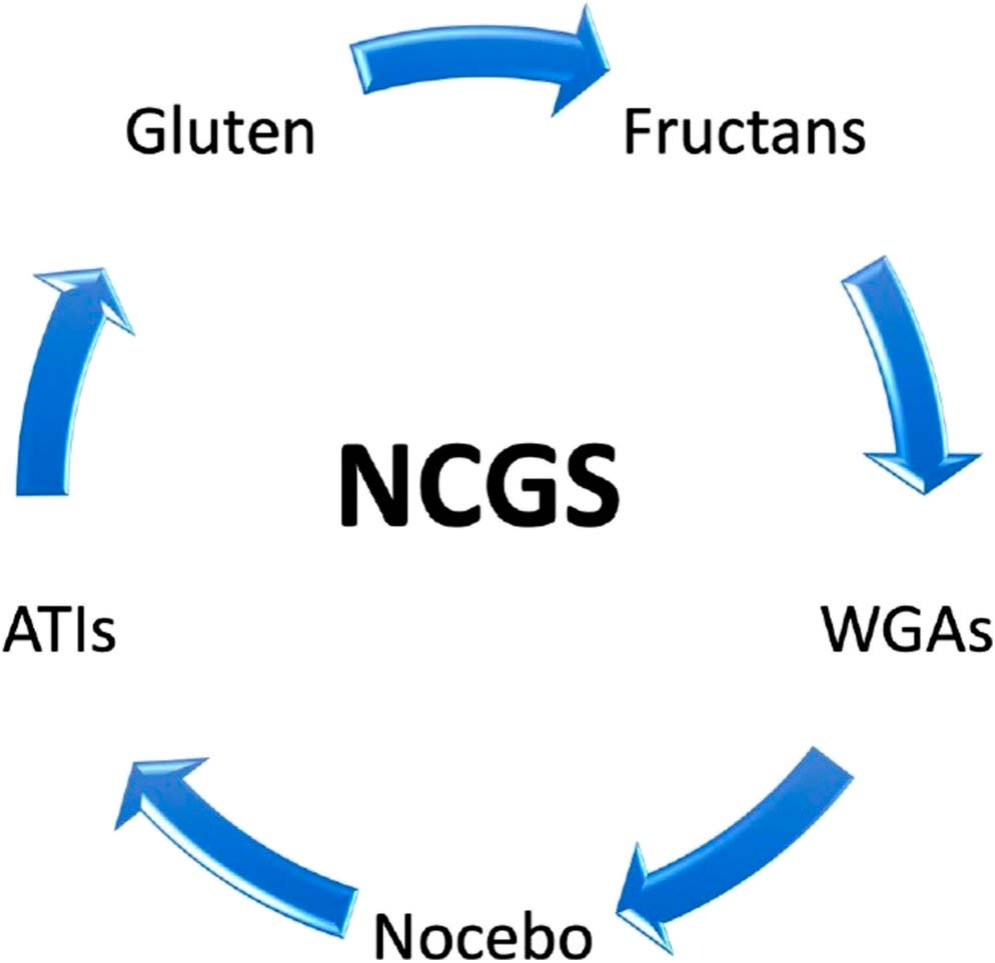
Potential pathophysiological mechanisms in NCGS. ATIs, alpha-amylase trypsin inhibitors; NCGS, nonceliac gluten sensitivity; WGA, wheat germ agglutinins.

Although certain gluten peptides, such as the α-gliadin peptide 31–43, may induce proinflammatory events in celiac disease (17,18) and may do so in NCG/WS (19), it is worth noting that other components of wheat may potentially be involved in the pathogenesis of NCG/WS. Fructans have been suggested to be responsible for the pathophysiology of NCG/WS, with a double-blind placebo-controlled challenge of 59 individuals demonstrating fructans, rather than gluten, inducing symptoms in self-reported NCG/WS (20). However, a recent study failed to note an association between fructan intake and gastrointestinal symptoms (21). Wheat germ agglutinins have also been postulated in the pathophysiology of NCG/WS, having been shown to alter enterocyte permeability *in vitro* (22) and being shown to stimulate proinflammatory cytokines (14,23). Similarly, alpha-amylase trypsin inhibitors have been shown to stimulate proinflammatory cytokines with subsequent intestinal inflammation (24), with further research required. Although these are all potential pathophysiological mechanisms, another alternative contributing to symptom generation could be a nocebo response (Figure 1).

### Diagnosis

It is essential that other gluten-related disorders are excluded before a diagnosis of NCG/WS. Although a large proportion of individuals presenting with gluten sensitivity will have NCG/WS, it is worth noting that up to 7% will have celiac disease (25). In view of this, celiac serology (immunoglobulin A [IgA]-endomysial antibodies or IgA-tissue transglutaminase antibodies) should be performed to exclude this diagnosis (while ensuring that the patient is on a normal, i.e., gluten-containing diet and is not IgA-deficient). In individuals who are unable to reintroduce gluten into their diet to test for celiac disease, HLA typing maybe of use. Negative HLA typing has a strong exclusion value for celiac disease, with between 97% and 99% of individuals with celiac disease having positive HLA typing (26,27). However, a positive result should be interpreted with caution, with up to 40% of the general population having a positive result (28). In addition, wheat allergy should be excluded before a diagnosis of NCG/WS. Currently, there is a lack of biomarkers for the diagnosis of NCG/WS, although a higher prevalence of antigliadin antibodies has been noted in this population, reported at around 50% (6). In addition, serum zonulin has been suggested as a biomarker for NCG/WS, with conflicting results (29,30). As a result, NCG/WS currently remains a clinical diagnosis based on assessing symptoms on a gluten-containing diet vs a GFD (8).

### Management

Like celiac disease, the cornerstone of management of NCG/WS remains a GFD (Table 1). However, uncertainties remain regarding the duration and threshold of dietary restriction required in NCG/WS. It is unclear whether individuals with NCG/WS should have a lifelong GFD, and it has been suggested that a trial of gluten reintroduction could be considered after 1–2 years (31). This seems to be a pragmatic approach because the GFD is not without risk, with potential nutritional inadequacies such as magnesium, selenium, fiber, iron, and calcium (32,33). Although this has been suggested, it has also been demonstrated that a large proportion of individuals with NCG/WS (64%) continue to follow a GFD at long-term follow-up (greater than 8 years), with symptom improvement seen in those strictly adherent to the diet (34). The threshold for gluten tolerance seems to be variable in individuals with NCG/WS, with the threshold required for symptom relief unknown (35). Ideally, the GFD should be implemented by a dietitian to prevent potential macronutrient and micronutrient inadequacy (36).

**Table 1.**
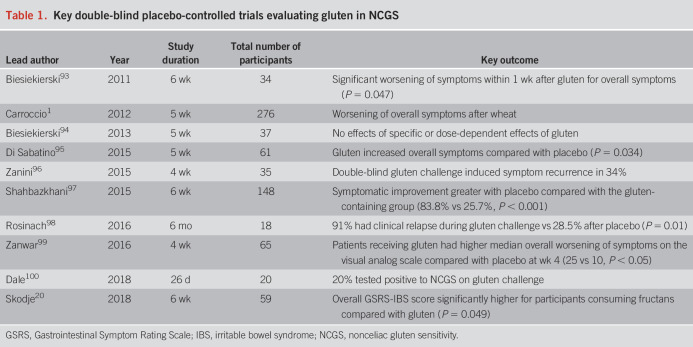
Key double-blind placebo-controlled trials evaluating gluten in NCGS

In addition, there seems to be a significant overlap between NCG/WS, IBS, and functional dyspepsia (37,38). It has previously been demonstrated that a large proportion of individuals with IBS have sensitivity to wheat, reported at between 23% and 49% (1,39,40). A key distinguishing feature between both IBS and NCGW/S is that individuals with NCG/WS tend to identify gluten as a trigger and self-report symptoms after the consumption of gluten. By contrast, individuals with IBS may only report this trigger when asked directly (19), but there is growing evidence that this group of patients may also respond effectively to a reduction in dietary gluten intake (41).

## EOSINOPHILIC GASTROENTERITIS

### Key concepts

Food intolerances take many forms, with some reactions not immune-mediated, such as those produced by enzyme deficiencies (lactase or sucrose-isomaltase deficiency) (42) or those induced by osmotic effects or fermentation of nonabsorbed carbohydrates, leading to enteric distension and symptoms in IBS. This concept is effectively targeted by the low FODMAP diet, which is now a standard of care for patients with IBS (43). The low FODMAP diet is a multiphasic diet, involving the reduction of all FODMAPs initially, followed by reintroduction of FODMAPs to tolerance and personalization subsequently (44).

Purely immune-driven reactions to food are more common in childhood and are typically IgE-mediated or mixed-type allergic reactions triggered by food ingestion (42), such as the rare eosinophilic gastrointestinal diseases. Eosinophils are found throughout the gastrointestinal tract where they perform a vital role in preserving mucosal immunity, especially from protozoal infections (45–47). Their presence distal to the esophagus is considered normal, although several primary gastrointestinal diseases are associated with increased numbers of eosinophils, including eosinophilic esophagitis (EoE), functional dyspepsia (primarily affecting the duodenum), and eosinophilic colitis (48). In small bowel disorders, EGE affects both the stomach and small bowel, with the role of diet in this condition explored below.

### Diagnosis

EGE is rare, with an estimated population prevalence of 5–8 per 100,000 people (49,50). A Th-2-type mucosal immune response is implicated in the pathogenesis of this disorder (3,51,52). It is defined by the presence of an abnormal number of eosinophils in the stomach or small bowel (although an exact cutoff is not agreed, one suggested has been greater than 52 eosinophils per high-powered field) (48,53).

EGE is associated with a wide range of nonspecific gastrointestinal symptoms, including abdominal pain, diarrhea, nausea, and vomiting, but can also present fulminantly, with surgical complications including perforation (49).

Food allergy confirmed by skin prick testing is common, overlapping with EGE in up to 44% of patients (3,52), further pointing toward food as an important driver of disease pathogenesis and shared etiology with EoE. However, the immune mechanism resulting from exposure to these food antigens in EGE is believed to be distinct from EoE, with a markedly different and distinct transcriptome (3).

### Management

Elimination and elemental diets have been shown to be efficacious in EGE (54,55). A systematic review including individual patient data for 86 patients reported dietary strategies to be effective in 88%, with the elemental diet, used in 29 children, leading to clinical remission in 76% (55). Similarly, a large case series of 17 children with EGE reported a clinical response rate to elimination diet strategies of 82% (54). However, it is worth noting that high-quality randomized studies are lacking, with further studies required. In addition, skin prick testing has not proven accurate in guiding dietary elimination strategies (55).

## SMALL INTESTINAL BACTERIAL OVERGROWTH

### Key concepts

Alterations of the gastrointestinal microbiome may play a role for a variety of gastrointestinal and extraintestinal conditions (56–60). Microbial “dysbiosis” is defined as alterations in the composition, density, and function of intestinal microbes. SIBO is an example of small intestinal dysbiosis. Although the conceptual framework of SIBO is now widely accepted, there is a gap in relation to generally accepted definitions of SIBO or universally established and accepted diagnostic criteria (61). SIBO remains a clinical disorder, presenting with a wide spectrum of symptoms ranging, typified by a microbial dysbiosis that is underpinned by abnormal microbial loads and/or abnormal types of microbes in these sites (62,63). The contaminating flora seen in the SIBO has featured both of oropharyngeal and colonic-type bacteria, but these occur in SIBO at different levels than their original location (63).

### Diagnosis

One of the fundamental problems in diagnosing SIBO is the lack of sensitive and specific and validated diagnostic tests. Several tests (culture-based or culture-independent) used to diagnose SIBO are outlined below, each having advantages and disadvantages (61).

### Direct test (aspirate/biopsy): qualitative and quantitative culture of proximal small bowel aspirates

Presence of ≥10^5^ colony-forming units per milliliter (CFU/mL) of colonic-type bacteria in the culture of jejunal aspirates is the traditionally accepted gold standard for diagnosing SIBO (64,65). However, bacterial concentrations of ≥10^5^ CFU/mL were mostly reported in initial studies investigating SIBO in patients with altered surgical anatomy (e.g., stagnant postsurgical loop syndrome). Healthy adults may have counts between 0 and 10^3^ CFU/mL, and more recently, a bacterial concentration of ≥10^3^ CFU/mL has become the cutoff criteria for diagnosing SIBO (66–68). Although the literature suggests sampling from the proximal jejunum, most physicians who perform luminal aspirations obtain samples from the duodenum using a standard upper endoscope where the concentration of bacteria is normally lower than the jejunum (69). In this context, a lower cutoff value of ≥10^3^ CFU/mL might be clinically more relevant for aspirations obtained from the proximal duodenum, given its proximal location, relative protection from translocation of bacteria from the colon, and its frequent exposure to acid from the stomach all of which would decrease risk of SIBO (70,71). However, aspiration and culture of small intestinal content have several limitations. It is an invasive, time-consuming, and technically challenging procedure, prone to cross-contamination by luminal and oropharyngeal contents, and lacks universal acceptance of optimal sampling site and cutoff thresholds for diagnosing SIBO.

An alternative approach to small intestinal aspiration is culturing biopsies obtained endoscopically from the small intestine. Because microorganisms are present in the mucus layer, which overlies the intestinal epithelium, culture from a mucosal biopsy is easier, faster, and more efficient to perform than aspiration. Although the mucosal biopsies are not inferior to the aspiration of small intestinal fluid (72,73), it is evident that the contamination of the working channel of the endoscope by microbes from the mouth, oropharynx, and the gastrointestinal tract ultimately will affect the sensitivity and specificity of biopsy-based tests or aspiration, unless precautions are taken to avoid cross-contamination. To address these methodological constraints, a novel aseptic biopsy device has been developed (the Brisbane Aseptic Biopsy Device, manufactured by MTW, Wesel, Germany), which allows mucosal biopsies to be obtained from the gastrointestinal tract without contamination by oral or luminal contents (74).

### Indirect (breath) tests

To overcome the limitations of the culture-dependent methods for diagnosing SIBO, indirect tests (breath tests) were developed. Quantification of hydrogen and methane gas in breath samples remains the most inexpensive, noninvasive, simple, and widely available test for diagnosing SIBO (67). Human cells are not capable of producing hydrogen or methane gas (75). Presence of these gases in the human breath indicates the metabolism of (nondigested) carbohydrates by gut microbes (76). Currently, the most used substrates are glucose and lactulose. The North American Consensus statement on hydrogen and methane breath testing (68) defines a rise over baseline of ≥20 parts per million for hydrogen by 90 minutes or a level of ≥ 10 parts per million in methane as a positive result consistent with the diagnosis of SIBO. Because more than one-third of healthy adult subjects are predominantly methane producers (77), it is important to measure both hydrogen and methane during breath tests. The recent American College of Gastroenterology guidelines has coined the term “intestinal methanogen overgrowth,” for emphasizing the importance of methane production by methanogens belonging to the domain *Archaea* rather than SIBO driven solely by bacteria (78). Hydrogen sulphide breath testing may be a potential biomarker for SIBO but requires validation (79).

However, breath tests also have several limitations. Overall, the sensitivity and specificity of breath tests for diagnosing SIBO are poor. Compared with the gold standard of small bowel aspiration and culture, the glucose breath test has a sensitivity of 62.5% and a specificity of 81.7%, whereas the lactulose breath test has a sensitivity of 52.4%–57.1% and a specificity of 84.6%–85.7% (80,81). Furthermore, there is a lack of consensus regarding the optimal substrate, doses of substrates, duration of the test, sampling intervals, and diagnostic thresholds (61).

Some of these shortcomings can be theoretically addressed by using gas-sensing capsules that measure luminal gas concentrations during transit of the small bowel. Preliminary studies have shown gas-sensing capsules were able to define regional fermentation patterns using hydrogen gas profiles (82). Hence, investigating the utility of the gas-sensing capsule as a means for “direct” assessment of microbial density presents an opportunity to overcome some of the shortcomings associated with the current breath test.

16S ribosomal RNA sequencing has demonstrated specific increases in the relative abundance of the phylum *Proteobacteria* in SIBO, with an altered proteobacterial profile that correlates with symptom severity, with further research required to explore this (81).

### Management

Changes of diet are the most basic intervention to modify the small intestinal microbiome. Although it could be speculated that a change of the diet will result in an adaptive change of the microbes colonizing the small intestine because of an altered microenvironment (4), other factors need to be taken into consideration. Fasting for several hours will convert a postprandial motility pattern to the interdigestive pattern that is characterized by the occurrence of propagated interdigestive motor complexes, associated with cyclic changes of gastric acid, bile, and pancreatic enzyme secretion (83,84).

It is well established that the somatostatin analog octreotide induces intestinal motor activity in healthy subjects or patients with motility disorders (85), whereas administration of erythromycin during the fasting state initiates propagated phase III contractions (86). Indeed, it has been shown that in patients with scleroderma and subsequent SIBO, at least short-term administration of octreotide reduces bacterial overgrowth while abdominal symptoms also improved (87). This demonstrates that treatments targeting small intestinal motility can have a beneficial effect in the setting of SIBO.

Changes in the composition of diet (e.g., a high- or low-gluten diet) also have been found to induce changes in the intestinal microbiome as reflected by fasting and postprandial hydrogen exhalation (88). Besides interventions that change the amount and composition of the diet, the use of antimicrobial agents is well established to treat patients with SIBO. In a systematic review published several years ago that included only 10 studies, antibiotics were more effective than placebo regarding normalization of breath tests (89), whereas the effects on gastrointestinal symptoms tended to correlate with breath test normalization. In a recent observational study, rotating antibiotic therapy with metronidazole and/or a quinolone (norfloxacin or ciprofloxacin) was superior to the use of a single agent (90). In recent years, several studies explored the effects of rifaximin on SIBO. A systematic review and meta-analysis, including 32 studies with 1,331 patients, found rifaximin to be effective and safe for the treatment of SIBO (91). However, the long-term response is poorly studied, and overall, the quality of evidence can be considered poor because of a number of limitations in the study designs. Although there are many studies assessing the effects of antimicrobial therapy in patients with IBS (56) and more recently in functional dyspepsia (57), recent data suggest that the clinical effects in patients presenting with upper abdominal symptoms are not influenced by concomitant symptoms of IBS (92).

For most patients, long-term improvement of symptoms is the objective of treatment, and very limited data are available on the longevity of any therapeutic interventions that is targeted to "normalize" the small intestinal dysbiosis. In conjunction with the obvious lack of a generally accepted gold standard for the diagnosis of SIBO (56), there are considerable knowledge gaps in relation to therapeutic measures that are intended to cure SIBO and provide long-lasting improvements to patients with this condition.

## CONCLUSION

Diet seems to play a key role in both the pathophysiology and management of small bowel disorders. Although diet seems to be a key trigger for symptoms in patients with NCGS, the component of wheat that triggers symptoms remains unclear. Although diet seems to be a key driver of disease pathogenesis in EGE, elimination and elemental diets remain to be properly validated in randomized, controlled trials.

Although diet may play a role in modifying the small intestinal microbiome in SIBO, it is worth noting that the evidence for using pharmacological treatments is currently greater. Further research is required to elucidate the role of diet in small bowel disorders in both pathophysiology and management.

## CONFLICTS OF INTEREST

**Guarantor of article:** David Surendran Sanders, MBChB, MRCP, MD, FACG, FRCP.

**Specific author contributions:** A.R., M.D.E.P., N.J.T., A.S., G.H., and D.S.S. drafted the initial manuscript. All authors reviewed and approved the final manuscript.

**Financial support:** G.H. and A.S.: National Health and Medical Research Council (APP1084544), Centre for Research Excellence (APP170993). D.S.S. and A.R.: None declared. N.J.T. and M.D.E.P.: National Health and Medical Research Council grants.

**Potential competing interests:** G.H. reports to be on the advisory boards Australian Biotherapeutics, Glutagen, and Bayer and received research support from Bayer, Abbott, Pfizer, Janssen, Takeda, and Allergan. He serves on the Boards of the West Moreton Hospital and Health Service, Queensland, UQ Healthcare, Brisbane, and the Gastro-Liga, Germany. He has a patent for the Brisbane aseptic biopsy device and serves as an editor of the Gastro-Liga Newsletter. D.S.S. receives an educational grant from Schaer (a gluten‐free food manufacturer). Dr. Schaer did not have any input in drafting of this manuscript. NJT reports nonfinancial support from HVN National Science Challenge NZ, personal fees from Aviro Health (Digestive health) (2019), Anatara Life Sciences, Brisbane (2019), Allakos (gastric eosinophilic disease) (2021), Bayer [IBS] (2020), Danone (Probiotic) (2018), Planet Innovation (Gas capsule IBS) (2020), Takeda, Japan (gastroparesis) (2019), twoXAR (2019) (IBS drugs), Viscera Labs (USA 2021) (IBS-diarrhea), Dr. Falk Pharma (2020) (EoE), Censa, Wellesley MA USA (2019) (Diabetic gastroparesis), Cadila PharmIncaceuticals (CME) (2019), Progenity Inc. San Diego (USA 2019) (Intestinal capsule), Sanofi-aventis, Sydney (2019) (Probiotic), Glutagen (2020) (Celiac disease), ARENA Pharmaceuticals (2019) (Abdominal pain), IsoThrive (2021) (esophageal microbiome), BluMaiden (2021), Rose Pharma (2021), Intrinsic Medicine (2021), and Comvita Mānuka Honey (2021) outside the submitted work; In addition, N.J.T. has a patent Nepean Dyspepsia Index (NDI) 1998, Biomarkers of IBS licensed, a patent Licensing Questionnaires Talley Bowel Disease Questionnaire licensed to Mayo/Talley, a patent Nestec European Patent licensed, and a patent Singapore Provisional Patent “Microbiota Modulation Of BDNF Tissue Repair Pathway” issued, “Diagnostic marker for functional gastrointestinal disorders” Australian Provisional Patent Application 2021901692. Committees: OzSage, Australian Medical Council (AMC) (Council Member); Australian Telehealth Integration Programme; MBS Review Taskforce; NHMRC Principal Committee (Research Committee) Asia Pacific Association of Medical Journal Editors. Boards: GESA Board Member, Sax Institute, Committees of the Presidents of Medical Colleges. Community group: Advisory Board, IFFGD (International Foundation for Functional GI Disorders), AusEE. Miscellaneous: Avant Foundation (judging of research grants). Editorial: Medical Journal of Australia (Editor in Chief), Up to Date (Section Editor), and Precision and Future Medicine, Sungkyunkwan University School of Medicine, South Korea, Med (Journal of Cell Press). N.J.T. is supported by funding from the National Health and Medical Research Council (NHMRC) to the Centre for Research Excellence in Digestive Health, and he holds an NHMRC Investigator grant. A.R., A.S., and M.D.E.P. declare no conflicts of interest.
